# Urologic surgery and invasive procedures during coronavirus pandemic: Retrospective comparison of risk infection in a referral Covid hospital and in a free-Covid hospital

**DOI:** 10.1177/0391560320927106

**Published:** 2020-05-10

**Authors:** Granata Antonio Maria, Petrainas Vasileios, Incarbone Giacomo Piero, Romanò Ai Ling Loredana, Rosso Marco, Garelli Giulia, Ranzoni Stefania, Marchesotti Federica, Andrea Gregori

**Affiliations:** Department of Urology, ASST FBF Sacco, Milan, Italy

**Keywords:** Covid-19, Sars-Cov-2, coronavirus, Italy, retrospective studies, emergencies, pandemic, urologists, spreading factor, Covid-19 prevention

## Abstract

**Introduction:**

On 21 February 2020, ‘Luigi Sacco’ Hospital was identified as a Covid-19 referral Hospital in Lombardy. The Department of Urology of our Healthcare Institution consists of two Urology Units, one at ‘L.Sacco’ Hospital (hereinafter referred to as Covid-19 hospital) and one at ‘Fatebenefratelli’ Hospital (hereinafter referred to as Covid-19-free hospital). The Healthcare System’s Administration communicated to all personnel the implementation of a planned ‘biological risk protocol’ at the Covid-19 hospital, while the Covid-19-free hospital followed regular government recommendations. We evaluated the risk of Sars-Cov-2 infection in the patients treated for surgical or invasive urologic procedures during the epidemic in the two different hospitals.

**Materials and methods:**

At the Covid-19 hospital, 12 patients underwent surgery and 51 patients invasive diagnostic procedures between 22 February and 8 March 2020; in the same period, at the Covid-19-free hospital, 21 patients underwent surgery and 56 patients invasive diagnostic procedures. We compared the incidence of Sars-Cov-2 infection among the patients who were accessed in the two Urology Units in the period of different restrictive measures after the outbreak of the epidemic.

**Results:**

We registered no cases of Sars-Cov-2 infection in the patients hospitalized in that period in Covid-19 hospital, despite three cases of swab positivity in the Covid-19-free hospital.

**Conclusion:**

The early implementation of extraordinary measures to restrict the spread of the virus offers a good protection also in a Covid-19 referral Hospital. The adoption of safety measures may be considered even after the end of the pandemic in all the health systems.

## Introduction

After the SARS epidemic (2003), ‘Luigi Sacco’ University Medical Center was identified as referral hospital of Northern Italy for activities regarding the biological risk of infective epidemics, caused by natural events or bioterrorism, which might represent serious hazards to the population.^1^ Therefore, dedicated pathways and areas were planned for the diagnosis and treatment of the patients affected by infectious diseases with high social impact; that was possible thanks to the contribution of the following Departments: Infectious Diseases, Microbiology, Emergency and Pathology. An ‘Infectious Emergency and Bioterrorism Unit’ was created in order to

- Coordinate all the procedures for emergencies like Sars or Ebola;
- Keep an update inventory of the equipment useful in case of crisis;
- Organize courses for health care professionals;
- Identify a 24-h available group of quick intervention in case of suspected bioterrorism (e.g. *envelope* filled with *anthrax*).

Under normal circumstances, ‘Luigi Sacco’ Hospital has two internationally recognized Departments of Infectious Diseases in a separate building with 75 recovery beds, 25 of which in negative pressure rooms ready to be used as intensive care rooms. The Intensive Care Unit (ICU) is provided with 17 recovery beds adjacent to the 12 operative theatres.

Coronavirus disease 2019 (Covid-19) is a clinical syndrome caused by a novel coronavirus called severe acute respiratory syndrome coronavirus 2 (Sars-Cov-2).

On the evening of 20 February, the first Italian Covid-19 case was diagnosed in Codogno’s hospital, in Lombardy region.^2^ On the night between 20th and 21st of February, ‘Luigi Sacco’s’ Infectious Emergency and Bioterrorism Unit was sent to Codogno’s hospital to evaluate the so-called ‘Italian patient 1’. On the morning of 21 February, we were informed that ‘Luigi Sacco’ Hospital was identified as a Covid-19 referral Hospital in Lombardy and thus all the planned procedures for an emergency were activated.

The 25 beds in the negative pressure rooms of the Infectious Diseases Department were freed up moving all the patients to other departments. The ICU adjacent to the main operative theatres was closed and transferred to the ground floor of the Infectious Disease Department and the ‘Covid-ICU’ became operational in few hours.

In the early afternoon, two patients were admitted to the Covid-ICU (Italian patient 1’s pregnant wife and one of his friends). During the weekend, the Infectious Disease Department only admitted Covid-19 patients coming from the whole Lombardy.

Since then, the Hospital Administration has been communicating to all health care personnel the implementation of a ‘biological risk protocol’ with the following measures to restrict the spread of the virus^3–7^:

- Dedicated pathways in the Emergency Department for patients with fever or respiratory symptoms (suspected Covid-19 cases);
- Proper use of individual protection devices (IPDs), like surgical masks, covers, gloves and so on for all doctors and nurses during the outpatient activities and for patients coming from outside;
- Recommended frequent hands washing with antiseptics;
- Specific droplet precautions with the use of filtering face piece masks (FFP2 or FFP3), water-repellent coats, safety visors/goggles, double gloves when visiting patients with clear or suspected respiratory syndrome, depending on the specific medical activity;
- Restriction of visitors for hospitalized patients;
- Measuring the body temperature of all the patients hospitalized for surgical and invasive procedures;
- Recommendations for patients’ transfer to the operating room (e.g. transfer through an exclusive pathway and elevator, wearing a surgical mask);
- Reduced activity of all surgical specialities with the suspension of non-urgent activities and the restrictions in scheduling the procedures that are non-deferrable or urgent, mainly owing the availability of anaesthesiologists who are engaged in the Covid-ICU.

The Department of Urology of our Healthcare Institution (Azienda Socio Sanitaria Territoriale ‘Fatebenefratelli-Sacco’) in Milan consists of two Urology Units, one at ‘Luigi Sacco’ University Medical Center (hereinafter referred to as Covid-19 hospital) and one at ‘Fatebenefratelli e Oftalmico’ Hospital (hereinafter referred to as Covid-19-free hospital), both led by the same Chief of Staff (A.G.). In both units, there are six Consultant Urologists.

During the first 15 days, after the first Italian case of Covid-19, the planned ‘biological risk protocol’ was immediately applied at the Covid-19 hospital, while the Covid-19-free hospital followed regular government recommendations.

## Materials and methods

We included in this report all the patients who underwent to surgical or invasive diagnostic procedures in the two urology Units between 22 February and 8 March.

At the Covid-19 hospital, 12 patients underwent a surgical procedure and 51 patients underwent invasive ambulatory procedures; in the same period, at the Covid-19-free hospital, 20 patients underwent a surgical procedure and 56 patients underwent invasive ambulatory procedures. Table 1 summarizes the procedures in the two hospitals.

**Table 1.** Procedures performed in the two hospitals du the evaluated period.

	Covid-19 hospital	Covid-19-free hospital
Surgical procedures		
LRP	2	3
LRC	2	1
LN	1	0
TURBT	4	8
ULT	0	6
Bilateral Nephrostomy	0	1
Ureteral Stenting	3	1
Invasive procedures		
Prostatic biopsies	8	10
Flexible Cystoscopies	32	33
Bladder instillation	11	13

LRP: laparoscopic radical prostatectomy; LRC: laparoscopic radical cystectomy; LN: laparoscopic nephrectomy; TURBT: transurethral resection of bladder tumour; ULT: ureterolithotripsy.

We evaluated the Sars-Cov-2 infection more than 15 days after the hospital discharge, considering that the average incubation period of COVID-19 is reported to be over 5 days and 97.5% of people who develop symptoms do so within 11.5 days from infection.^8^

Between 23 and 27 March, we contacted by phone all the patients treated with surgical or invasive procedures in the two Urology Units during the period when different restriction measures were adopted by the two hospitals.

We interviewed the patients in order to

- Assess their health status after the urological procedures;
- To find out about possible respiratory symptoms, fever and an eventual diagnosis of infection by Sars-Cov-2, appeared after hospital discharge.

The aim of the evaluation was to compare the risk of infection between a Covid-19 hospital adopting a strict biological risk protocol and a theoretically Covid-19-free hospital adopting standard safety procedures.

## Results

No cases of Sars-Cov-2 infection were registered among the 63 patients who were accessed in the selected period in the Urology Unit of the Covid-19 hospital. All patients were free of symptoms attributed to Sars-Cov-2 infection (fever, coughing, dyspnoea, diarrhoea, conjunctivitis, taste or olfactory disorders)^9,10^; consequently, none of them were swab-tested, because swabs are only used on symptomatic patients according to the regional diagnostic policy.

Among the 76 patients hospitalized in the Covid-19-free hospital, 3 patients were positive for Sars-Cov-2 infection.

Two cases had mild symptoms and were sent home in quarantine, with active surveillance. They underwent bilateral nephrostomy for advanced bladder cancer and ureterolithotripsy for complicated renal colic, 6 and 9 days before the Sars-Cov-2 positivity, respectively. One patient, who underwent transurethral resection of a bladder tumour was re-hospitalized 11 days after discharge with a diagnosis of Covid-19 pneumonia. Table 2 summarizes the results.

**Table 2.** Sars-Cov-2 infections in the study population.

Urology department	Surgical procedure	Invasive procedures	Total patients 23–27 March	Positive for Sars-Cov-2 infection	Mild symptoms	Pneumonia
Covid-19 hospital	12	51	63	0	0	0
Covid-19-free hospital	20	56	76	3	2	1

Finally, we registered two cases of Sars-Cov-2 infection among the urologists from the Covid-19-free hospital (one is in quarantine after fever episodes and a positive nasal swab test while the other was admitted in hospital with Covid-19 pneumonia).

## Discussion

Today, all the north of Italy, in particular the Lombardy region, is fighting a real war against a terrible enemy. The medical specializations gradually lost importance, because in several hospitals like ours, due to the shortage of specific specialists, such as infectious diseases’ specialist, pneumologist and anaesthesiologist, Covid-19 patients are managed also by other specialists such as general surgeons, urologists, orthopaedics, ophthalmologists and so on.

When the virus out broke in northern Italy, probably the health care system underestimated the risks, and the initial idea of the local governments was to concentrate the Covid-19 cases in just few hospitals. A few weeks later, due to the outburst of the number of patients needing hospitalization and intensive care, it was clear in Lombardy region that all public and private hospitals had to play a central role in the management of the epidemic.

We were wondering if the access to a Covid-19 hospital represented a severe risk of infection for patients hospitalized for other pathologies. We expected a higher rate of Sars-Cov-2 infection in patients who entered the Covid-19 hospital, than in those who entered the Covid-19-free hospital.

Conversely, our evaluation indicates that we had more infections in a Covid-19-free hospital, with a late implementation of the security protocols. In fact, patients operated in the Covid-19-free hospital underwent also deferrable procedures, did not wear surgical masks during the hospitalization and the transportation to the operating theatre, and had fewer limitations for visitors.

That may suggest that the early adherence to strict safety protocols and the correct use of IPDs can guarantee a safer pathway for the patients being hospitalized for urologic problems, even in a Covid-19 referral hospital.

Moreover, a lack of preparedness within the health care settings may be considered a factor promoting superspreading events.^11^

Possible biases of this report may be represented by the low number of patients and the impossibility to demonstrate that Sars-Cov-2 was contracted during hospital recovery.

However, in our opinion, it is mandatory to start defining protocols for all the patients who will enter hospitals in the next future mainly for two reasons:

- First, hospitals can become an important spreading factor of the epidemic, especially in many countries still not following World Health Organisation’s recommendations on containment;
- Second, there is no defined scientific prediction to the end of the pandemic, and therefore it is necessary to restart a normal surgical activity, also with elective procedures, trying to guarantee patients a low infective risk during the hospital stay.

## Conclusion

Relying on a wider range of cases with the design of surveillance models could ensure safer pathways for all patients accessing hospitals, especially for those hospitals not directly involved in the treatment of Covid-19. This may improve our preparedness to fight the Sars-Cov-2, as well as future infectious disease outbreaks.

## References

[1] Ministero della Salute. Piano Sanitario 2003-2005 (GU Serie Generale n.139 del 18–06–2003 – Suppl. Ordinaries n. 95), http://www.salute.gov.it/imgs/C_17_pubblicazioni_654_allegato.pdf

[2] Gagliano A Villani PG Co F, et al COVID-19 epidemic in the middle province of northern Italy: impact, logistic and strategy in the first line hospital. Disaster Med Public Health Prep 2020 Epub ahead of print 24 March 2020. DOI: 10.1017/dmp.2020.51. PMC7156580 32207676

[3] Weber DJ Sickbert-Bennett E Gergen MF, et al Efficacy of selected hand hygiene agents used to remove *Bacillus atrophaeus* (a surrogate of *Bacillus anthracis*) from contaminated hands. JAMA 2003; 289(10): 1274–1277. 12633189 10.1001/jama.289.10.1274

[4] Loeb M McGeer A Henry B, et al SARS among critical care nurses, Toronto. Emerg Infect Dis 2004; 10(2): 251–255. 15030692 10.3201/eid1002.030838 PMC3322898

[5] Fowler RA Guest CB Lapinsky SE, et al Transmission of severe acute respiratory syndrome during intubation and mechanical ventilation. Am J Respir Crit Care Med 2004; 169(11): 1198–1202. 14990393 10.1164/rccm.200305-715OC

[6] Srinivasan A McDonald LC Jernigan D, et al Foundations of the severe acute respiratory syndrome preparedness and response plan for healthcare facilities. Infect Control Hosp Epidemiol 2004; 25(12): 1020–1025. 15636287 10.1086/502338

[7] Park J Yoo SY Ko JH, et al Infection prevention measures for surgical procedures during a Middle East Respiratory Syndrome outbreak in a tertiary care hospital in South Korea. Sci Rep 2020; 10(1): 325. 31941957 10.1038/s41598-019-57216-x PMC6962363

[8] Lauer SA Grantz KH Bi Q, et al The incubation period of coronavirus disease 2019 (COVID-19): from publicly reported confirmed cases: estimation and application. Ann Intern Med 2020; 172(9): 577–582. 32150748 10.7326/M20-0504 PMC7081172

[9] Huang C Wang Y Li X. Clinical features of patients infected with 2019 novel coronavirus in Wuhan, China. Lancet 2020; 395(10223): 497–506. 31986264 10.1016/S0140-6736(20)30183-5 PMC7159299

[10] Giacomelli A Pezzati L Conti F, et al Self-reported olfactory and taste disorders in SARS-CoV-2 patients: a cross-sectional study. Clin Infect Dis 2020; 71(15): 889–890. 32215618 10.1093/cid/ciaa330 PMC7184514

[11] Frieden TR Lee CT. Identifying and interrupting superspreading events: Implications for control of severe acute respiratory syndrome coronavirus 2. Emerg Infect Dis 2020; 26(6): 1059–1066. 32187007 10.3201/eid2606.200495 PMC7258476

